# Enhancing the Antipsychotic Effect of Risperidone by Increasing Its Binding Affinity to Serotonin Receptor via Picric Acid: A Molecular Dynamics Simulation

**DOI:** 10.3390/ph15030285

**Published:** 2022-02-24

**Authors:** Majid Alhomrani, Walaa F. Alsanie, Abdulhakeem S. Alamri, Hussain Alyami, Hamza Habeeballah, Heba A. Alkhatabi, Raed I. Felimban, John M. Haynes, Sonam Shakya, Bassem M. Raafat, Moamen S. Refat, Ahmed Gaber

**Affiliations:** 1 Department of Clinical Laboratories Sciences, The Faculty of Applied Medical Sciences, Taif University, P.O. Box 11099, Taif 21944, Saudi Arabia; m.alhomrani@tu.edu.sa (M.A.); w.alsanie@tu.edu.sa (W.F.A.); a.alamri@tu.edu.sa (A.S.A.); 2 Centre of Biomedical Sciences Research (CBSR), Deanship of Scientific Research, Taif University, P.O. Box 11099, Taif 21944, Saudi Arabia; 3 College of Medicine, Taif University, P.O. Box 11099, Taif 21944, Saudi Arabia; hmyami@tu.edu.sa; 4 Faculty of Applied Medical Sciences in Rabigh, King Abdulaziz University, Jeddah 21589, Saudi Arabia; hhabeeballah@kau.edu.sa; 5 Department of Medical Laboratory Sciences, Faculty of Applied Medical Sciences, King Abdulaziz University, Jeddah 21589, Saudi Arabia; halkhattabi@kau.edu.sa (H.A.A.); faraed@kau.edu.sa (R.I.F.); 6 Center of Excellence in Genomic Medicine Research (CEGMR), King Abdulaziz University, Jeddah 21589, Saudi Arabia; 7 Hematology Research Unit, King Fahd Medical Research Centre, King Abdulaziz University, Jeddah 21589, Saudi Arabia; 8 Center of Innovation in Personalized Medicine (CIPM), 3D Bioprinting Unit, King Abdulaziz University, Jeddah 21589, Saudi Arabia; 9 Monash Institute of Pharmaceutical Sciences, 381 Royal Parade Parkville, Melbourne, VIC 3052, Australia; john.haynes@monash.edu; 10 Department of Chemistry, Faculty of Science, Aligarh Muslim University, Aligarh 202002, India; sonamshakya08@gmail.com; 11 Department of Radiological Sciences, College of Applied Medical Sciences, Taif University, P.O. Box 11099, Taif 21944, Saudi Arabia; bassemraafat@tu.edu.sa; 12 Department of Chemistry, College of Science, Taif University, P.O. Box 11099, Taif 21944, Saudi Arabia; 13 Department of Biology, College of Science, Taif University, P.O. Box 11099, Taif 21944, Saudi Arabia

**Keywords:** risperidone, charge-transfer complexes, antipsychotic drug, molecular docking

## Abstract

The aim of this study was to assess the utility of inexpensive techniques in evaluating the interactions of risperidone (Ris) with different traditional π-acceptors, with subsequent application of the findings into a Ris pharmaceutical formulation with improved therapeutic properties. Molecular docking calculations were performed using Ris and its different charge-transfer complexes (CT) with picric acid (PA), 2,3-dichloro-5,6-dicyanop-benzoquinon (DDQ), tetracyanoquinodimethane (TCNQ), tetracyano ethylene (TCNE), tetrabromo-pquinon (BL), and tetrachloro-p-quinon (CL), as donors, and three receptors (serotonin, dopamine, and adrenergic) as acceptors to study the comparative interactions among them. To refine the docking results and further investigate the molecular processes of receptor–ligand interactions, a molecular dynamics simulation was run with output obtained from AutoDock Vina. Among all investigated complexes, the [(Ris) (PA)]-serotonin (CTcS) complex showed the highest binding energy. Molecular dynamics simulation of the 100 ns run revealed that both the Ris-serotonin (RisS) and CTcS complexes had a stable conformation; however, the CTcS complex was more stable.

## 1. Introduction

Risperidone (Ris) is a second-generation antipsychotic that has been used to treat psychotic disorders, including schizophrenia, since the 1990s [1,2]. Compared to first-generation antipsychotics such as haloperidol, Ris is less likely to cause extrapyramidal side effects and is thereby frequently prescribed in clinical practice [1]. In addition, Ris has the added value of reducing undesirable symptoms associated with schizophrenia, such as social withdrawal and lack of motivation [2].

According to the literature, it being a safe, effective, and tolerable molecule categorized it into the World Health Organization’s List of Essential Medicines [3]. Moreover, the use of Ris extends beyond the treatment of schizophrenia to the treatment or management of other psychiatric conditions, such as mood disorders and behavioral symptoms associated with autism [3,4]. Furthermore, patients suffering from acute psychosis have a high prevalence of comorbid depression in up to 75% of the cases [5]. Given such a high prevalence of depression among patients with schizophrenia, treatment goals includes targeting multiple receptors responsible for such conditions. While dopaminergic receptors are responsible for schizophrenia [6], serotonergic receptors are responsible for depressive disorders [7].

Ris is an antagonist for a number of receptors, including dopaminergic (D1, D2), serotonergic (of 5-HT2A), and adrenergic (α1, α2) receptors. Its high affinity to 5-HT2A in comparison to D2 is behind its beneficial effects in ameliorating the undesirable symptoms of schizophrenia [8,9]. Furthermore, its tolerability is due to its low affinity for dopamine receptors, which is less than that of the first-generation antipsychotic haloperidol [8]. These findings justify the rationale for targeting Ris-related receptors as a possible option for the clinical improvement of symptoms associated with schizophrenia [9,10]. This is very crucial in designing a drug with a multireceptor profile in order to optimize its therapeutic effects for comorbid conditions such as schizophrenia and depression [11].

In biochemical and bioelectrochemical energy transfer processes, donor–acceptor interactions are critical and significant [12]. The formation of charge transfer (CT) complexes with some p-acceptors was extensively explored spectrophotometrically for the efficacy of medicines [13,14,15]. In many chemical processes, such as addition, substitution, and condensation, the interactions of charge-transfer complexes are well-recognized [16].

CT interactions between electron donors and acceptors are also crucial in drug–receptor binding mechanisms [17], surface chemistry [18], and many biological domains [17]. In addition, the CT reactions of p-acceptors were successfully used in pharmacological studies [19] and in the determination of electrochemical properties [20,21].

In this study, Autodock Vina was used for molecular docking to study the interactions between the ligand (Ris and synthesized CT complexes) and receptors (serotonin, dopamine, and adrenergic receptors). Binding energies, along with hydrophobic and hydrogen bond surface interactions, were also determined. To provide a more effective mechanism for demonstrating receptor–ligand interactions, the best molecular docking data were subjected to a molecular dynamics simulation at 300 K for 100 ns. This kind of modelling is endorsed in the literature for further understanding and enhancing the therapeutic benefits of such antipsychotics [8]. In terms of residue flexibility, the dynamic features of the complexes were compared in terms of structural stability, solvent-accessible surface area, structure compactness, and hydrogen bond interactions. The addition of picric acid is likely to affect the binding affinity of Ris to the relevant multiple receptors, which is likely to enhance its therapeutic action.

## 2. Results and Discussion

### 2.1. Preface of Six-Risperidone Solid Charge Transfer Complexes

**Figure 1:**
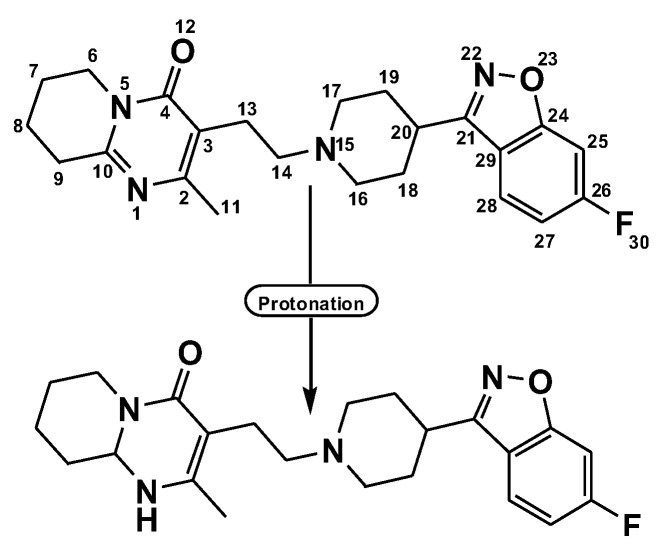
Proton transfer of risperidone drug.

Because Ris has many electron density sites, it could be a good electron donor. After the protonation of N(1)-H [22], the presence of a pyrimidine ring in the structure of Ris (Figure 1) works as a base and *n*-donor to form a charge transfer complex with π-acceptors.

**Figure 2:**
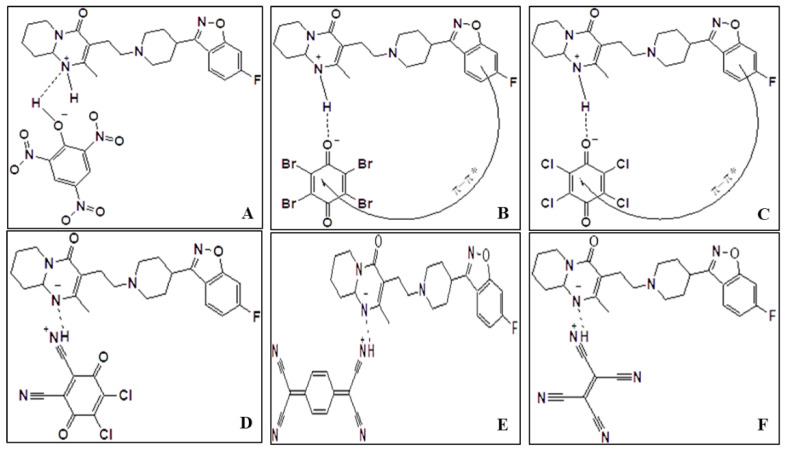
Speculated formula of Ris-PA (**A**), Ris-BL (**B**), Ris-p-CL (**C**), Ris-DDQ (**D**), Ris-TCNQ (**E**), and Ris-TCNE (**F**) charge transfer complexes.

According to the generation of positive and negative dative anions under donor–acceptor chelation (Figure 2), the conductance values show that the charge transfer complexes are slightly electrolytic [19]. We had characterized all these charge-transfer complexes using infrared, Raman, and 1H NMR spectra, and X-ray powder diffraction (XRD) [23].

The way of charge carriers between valence and conduction bands determines the absorption process of photons in charge transfer. The band structure concept, which is utilized in semiconductor electronic transitions, was adapted to optical absorption in organic systems. The highest occupied molecular orbital (HOMO; π-orbital) contributes to the valence band of a molecular crystal, whereas the lowest unoccupied molecular orbital (LUMO; π*-orbitals) contributes to the conduction band [23].

The band gap (Eg), which can be calculated from variations in optical absorption at the basic absorption edge, separates these bands. The following Bardeen formula [24] can be used to describe the relationship between absorption coefficients as a function of photon energy:

αhν = C(hν − E_g_)^n^ (1)

where α is obtained from formula α(ν) = 2.303 A/d, A is absorbance, and d is the thickness of the polymer film [25].

**Figure 3:**
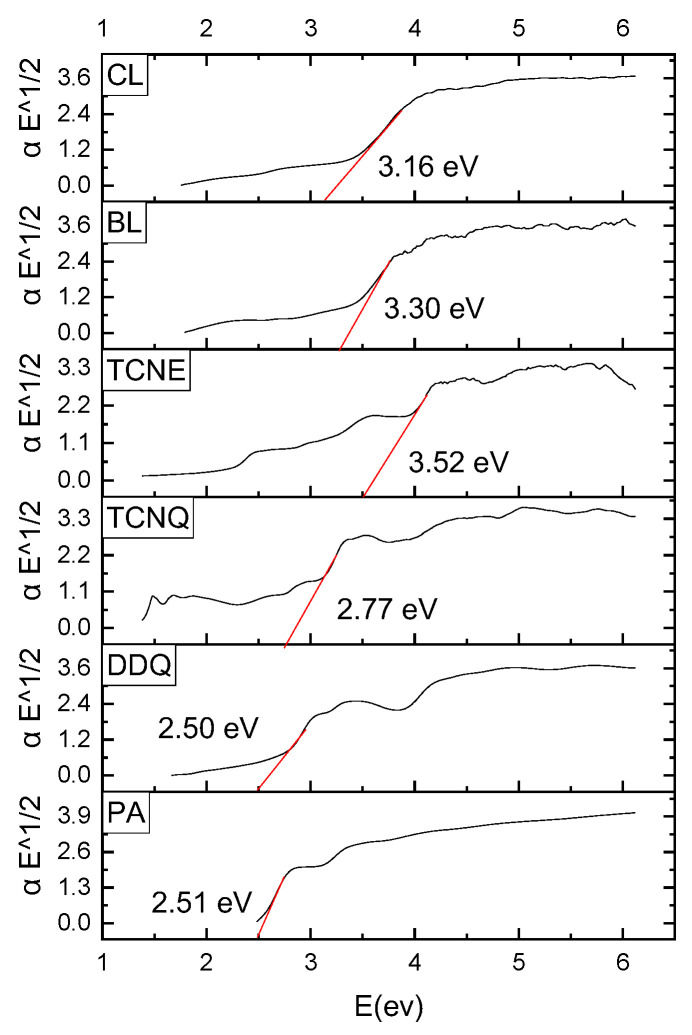
Plots of (αhν)^1/2^ as a function of photon energy for UV absorption of six-risperidone solid charge transfer complexes.

Constant C is a transition probability-dependent parameter. The direct (*n* = 1/2) and indirect (*n* = 2) permissible transitions are defined by the value of the constant n. (αhv)^1/2^ was plotted as a function of hν using the indirect transition (*n* = 2) as shown in Figure 3. Extrapolating the linear component of the obtained curves to zero absorption yields optical band gap E_g_. The probability of transition decreases as the number of charge carriers on localized states increases, necessitating more absorption in these locations, thereby narrowing the band gap [26].

### 2.2. Molecular Docking

Synthesized CT complexes, viz. [(Ris) (PA)], [(Ris) (DDQ)], [(Ris) (TCNQ)], [(Ris) (TCNE)], [(Ris) (BL)], and [(Ris) (CL)] were docked against serotonin (PDB ID: 6BQH), dopamine (PDB ID: 6CM4), and adrenergic (PDB ID: 6KUW) receptors, and the best docking poses obtained. Ris (donor moiety) was used as a control for comparative purposes. Molecular docking of the aforementioned six CT complexes revealed that their potential binding energy was higher than that of Ris at all receptors (Table 1).

**Figure 4:**
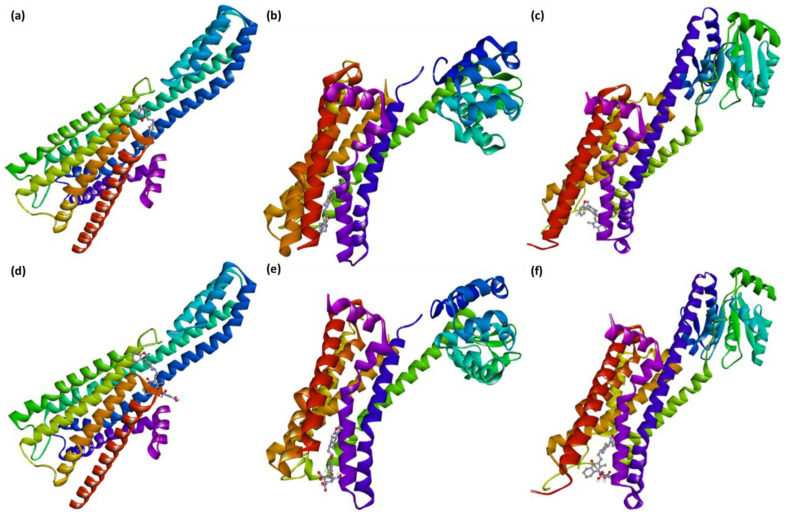
Best docked pose showing a helical model of serotonin, dopamine, and adrenergic receptors docked with (**a**–**c**) risperidone and (**d**–**f**) CTcS complexes.

Among the six CT complexes screened, [(Ris) (PA)] showed the highest docking energy compared to Ris. Molecular docking of [(Ris) (PA)] with serotonin, dopamine, and adrenergic receptors revealed potential binding energies of −11.4, −10.6, and −10.2 kcal/mol, respectively. The highest binding energy value of [(Ris) (PA)]-serotonin (CTcS) signifies a stronger interaction than that between dopamine and adrenergic receptors. The interactions between [(Ris) (PA)] and the CTcS complex with the receptors are depicted in Figure 4, with docking and interaction data presented in Table 2 and Table 3.

**Figure 5:**
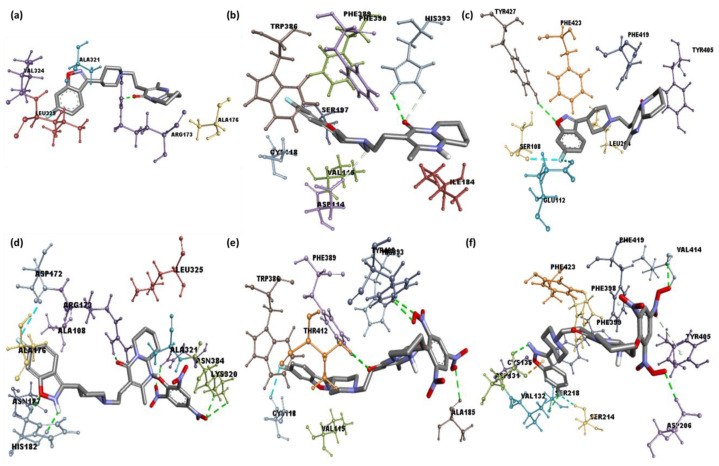
3D representations of serotonin, dopamine, and adrenergic receptors docked with (**a**–**c**) risperidone and (**d**–**f**) CTcS complex.

**Figure 6:**
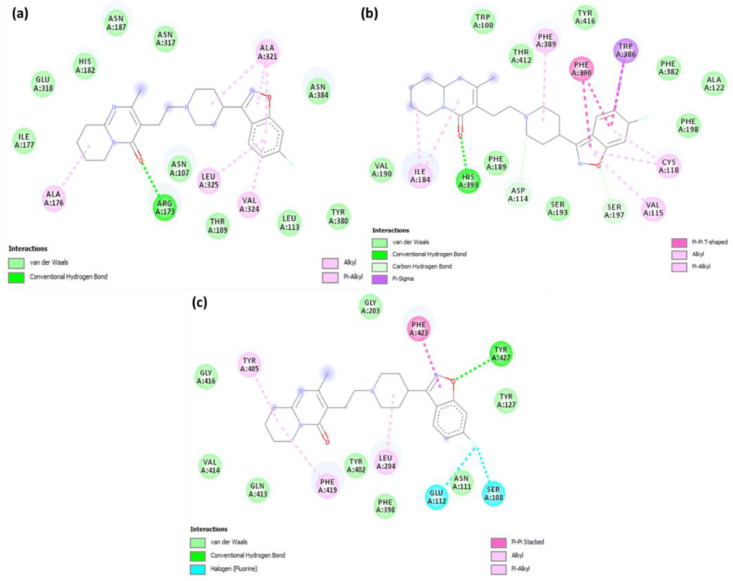
2D representations of interactions between risperidone and (**a**) serotonin, (**b**) dopamine, and (**c**) adrenergic receptors.

**Figure 7:**
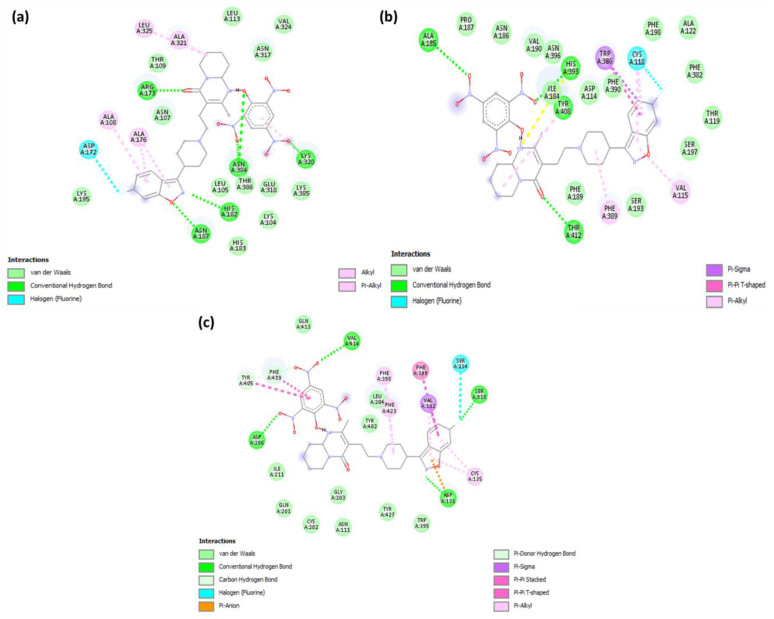
2D representations of interactions between CTcS complex and (**a**) serotonin, (**b**) dopamine, and (**c**) adrenergic receptors.

**Figure 8:**
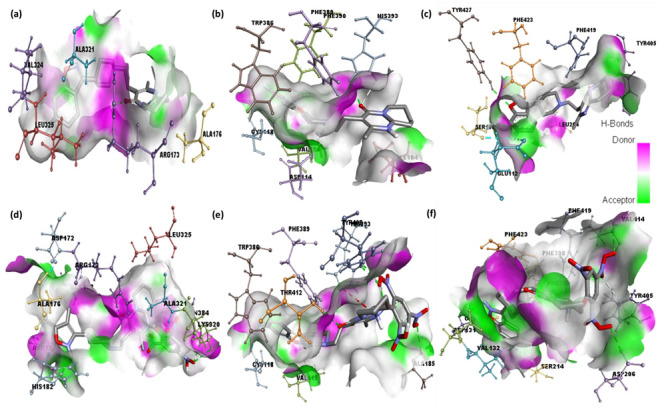
Representations of hydrogen bonding surfaces of serotonin, dopamine, and adrenergic receptors docked with (**a**–**c**) risperidone and (**d**–**f**) CTcS complex.

**Figure 9:**
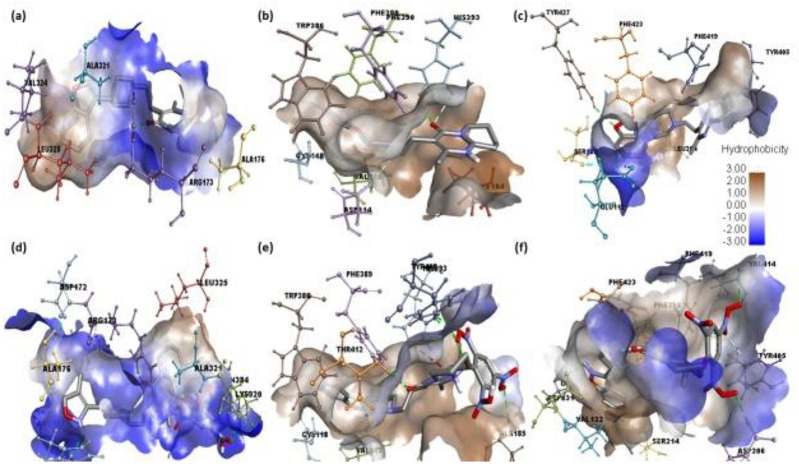
Representations of hydrophobic binding surfaces of serotonin, dopamine, and adrenergic receptors docked with (**a**–**c**) risperidone and (**d**–**f**) CTcS complex.

Analysis of the best-docked pose of [(Ris) (PA)]-serotonin revealed that the amino acid residues, including His182, Asn187, Asn384, Lys320, and Arg173, formed hydrogen bond interactions. In addition, Leu325, Ala321, Ala108, and Ala176 established π-alkyl interactions while Asp172 formed a halogen (fluorine) interaction [27,28]. The best-docked pose of [(Ris) (PA)]-dopamine revealed that the amino acid residues, including Thr142, Ala185, His393, and Tyr408, formed hydrogen bond interactions, Val115 and Phe389 established π-alkyl interactions, Trp386 established π-sigma, and Cys118 formed a halogen (fluorine) interaction [29,30]. The best-docked pose of the [(Ris) (PA)]-adrenergic receptor interaction revealed that the amino acid residues Val414, Asp206, Asp131, and Ser218 formed hydrogen bond interactions, while Phe398, Phe423, and Cys135 established π-alkyl interactions, and Val132 and Ser214 established π-sigma and halogen (fluorine) interactions, respectively. Similarly, molecular docking of Ris with serotonin, dopamine, and adrenergic receptors revealed potential binding energies of −9.6, −8.4, and −9.1 kcal/mol, respectively. The higher binding energy value of Ris with serotonin (RisS) signifies a stronger interaction than that of dopamine and adrenergic receptors. These data show that the CT complex ((Ris) (PA)) binds with all three receptors more efficiently than the reactant donor (Ris) does; among all complexes, the CTcS complex showed the highest binding energy. 3D representations of the interactions between Ris and the CTcS complex with the investigated receptors are shown in Figure 5, while the 2D representations are shown in Figure 6 and Figure 7. In addition, the surfaces of the hydrophobic and hydrogen bond interactions are shown in Figure 8 and Figure 9.

### 2.3. Molecular Dynamics Simulation

**Figure 10:**
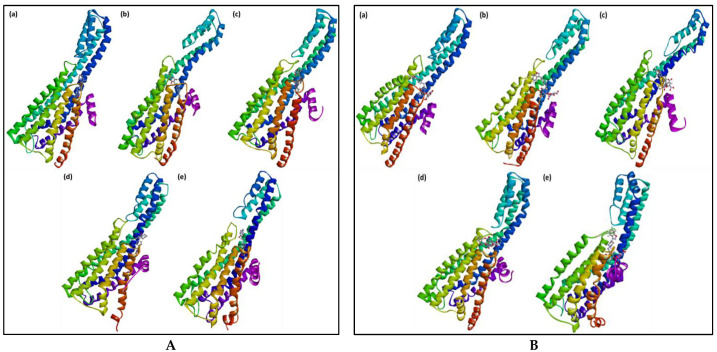
RisS structures (**A**) and CTcS structures (**B**) at (**a**) 1 ns, (**b**) 10 ns, (**c**) 20 ns, (**d**) 50 ns, and (**e**) 100 ns molecular dynamics runs, giving a visual representation of event sequence and process dynamics.

The best-docked pose (RisS and CTcS) data with the highest docking score generated from AutoDock Vina was utilized as the starting structure for the 100 ns molecular dynamics (MD) simulation run. Only the best-docking output was employed to build up this method in a high-throughput manner to study the binding mechanism of the ligand at the active site of the protein under clearly defined water environments. The different structures represented in Figure 10 give a visual representation of the sequence of events and the dynamics of the process during the 1, 10, 20, 50, and 100 ns production runs.

**Figure 11:**
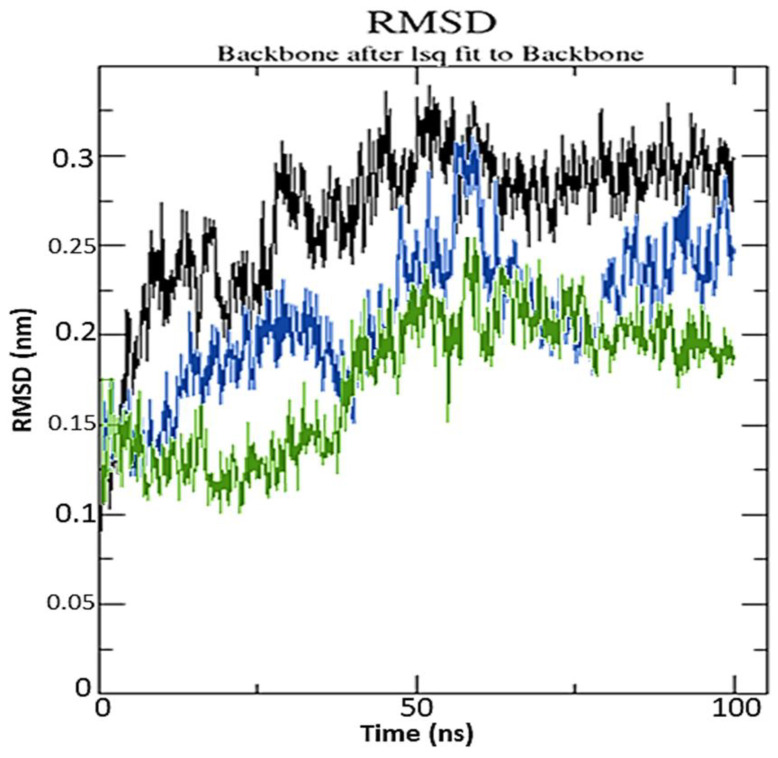
Root mean square deviation of solvated receptor backbone and ligand complex during the 100 ns molecular dynamics simulation: RisS complex (blue) and CTcS complex (green), and unbound serotonin receptor (black).

To examine structural stability, MD data were processed by calculating the root mean square deviation (RMSD). RisS and CTcS formed stable conformations after ~75 and ~62 ns, respectively, with RMSD values of 2.61 and 2.21 Å, respectively, as seen in the RMSD plot (Figure 11).

**Figure 12:**
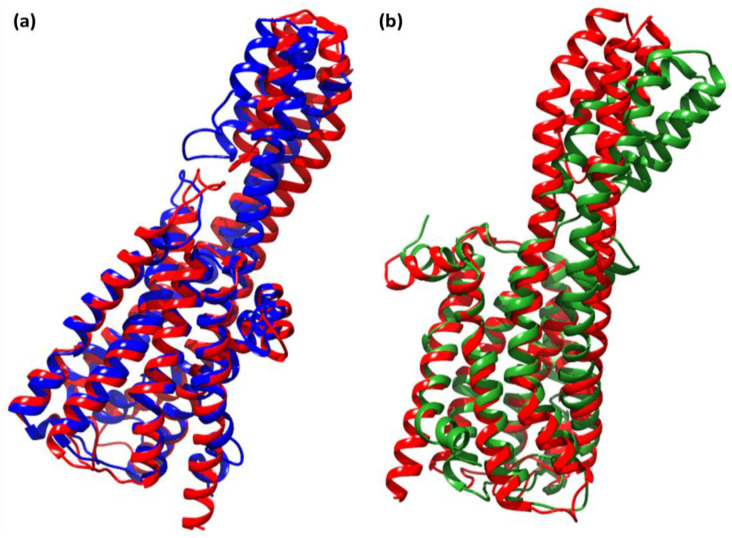
Superimposed structures of (**a**) unbound serotonin receptor (red) and serotonin receptor after simulation (blue) for the RisS complex, and (**b**) unbound serotonin receptor (red) and serotonin receptor after simulation (green) for the CTcS complex.

The most acceptable RMSD value range is <3.0 Å, as low RMSD values indicate superior stability of the system [31]. Our findings show that the CTcS complex developed a more stable combination than the RisS complex did. The low RMSD values of RisS and CTcS reflects a conformational alteration in the protein secondary structure due to ligand binding. The findings show that ligand-receptor interactions bring protein chains closer and reduce the gap between them (Figure 12) [32,33].

**Figure 13:**
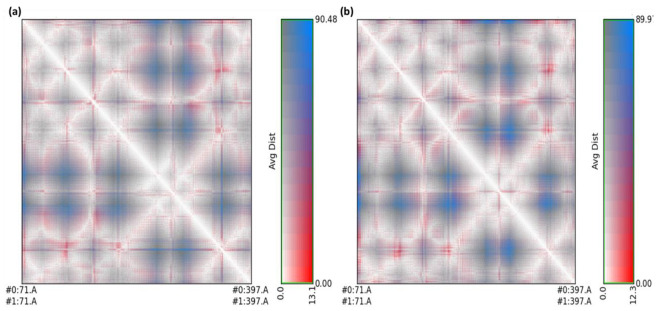
RR distance map displaying patterns of spatial interactions between (**a**) unbound serotonin receptor and serotonin receptor after simulation for RisS, and (**b**) unbound serotonin receptor and serotonin receptor after simulation for CTcS, showing average distance and standard deviation for all amino acid pairs.

**Figure 14:**
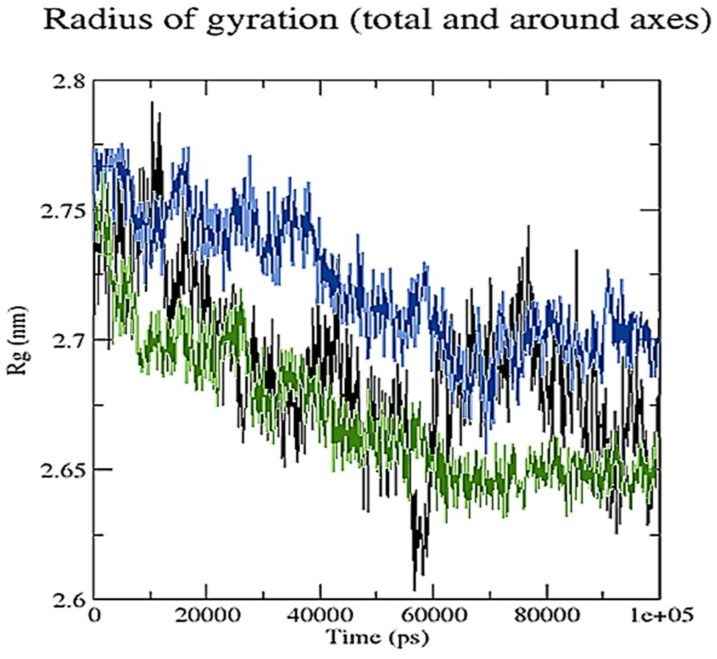
Radius of gyration for unbound serotonin receptor (black), RisS complex (blue), and CTcS complex (green) during the 100 ns simulation period.

To evaluate and compare protein structures, RR distance maps (two-dimensional representations of protein 3D structure) representing the average distance and standard deviation for all amino acid pairings between two conformations are employed [34]. The RR distance maps (Figure 13) elucidate patterns of spatial interactions [35,36]. The white diagonal on the map shows the zero distance between two residues, while the red and blue elements represent residue pairings with the greatest distance variances in the two conformations. Average radius of gyration (Rg) values of 27.38 and 26.53 Å were observed for RisS and CTcS, respectively. During the simulation, the Rg values for RisS and CTcS decreased, indicating that the structures became more compact (Figure 14).

**Figure 15:**
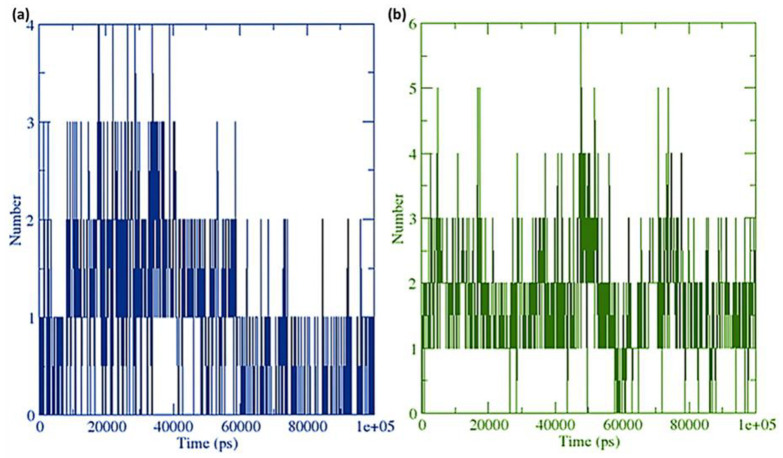
Number of average hydrogen bonding interactions between (**a**) the RisS complex and (**b**) the CTcS complex during the 100 ns simulation period.

**Figure 16:**
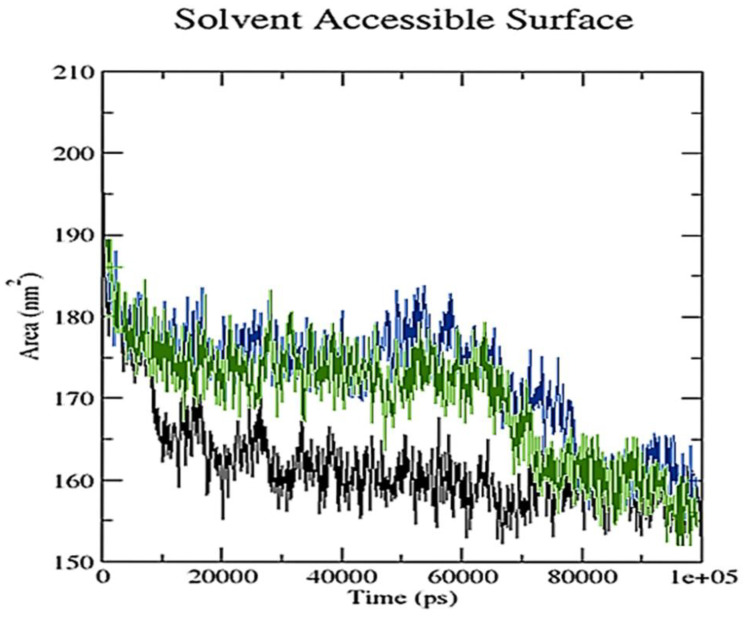
Solvent accessible surface area analysis for unbound serotonin receptor (black), the RisS complex (blue), and the CTcS complex (green) during the 100 ns simulation period.

A grid-search on 25 × 11 × 14 grids, rcut = 0.35, revealed the number of hydrogen bond interactions between ligand and receptor combinations (RisS and CTcS), which were plotted against time (Figure 15). When calculating hydrogen bonds between the ligand (34 and 52 atoms for RisS and CTcS complexes, respectively) and receptor (3706 atoms), 508 donors for both RisS and CTcS complexes, and 990 and 1000 acceptors for RisS and CTcS complexes, respectively, were observed. The average numbers of hydrogen bonds per timeframe were observed to be 0.937 and 1.709 out of 251,460 and 254,000 possible outcomes for RisS and CTcS, respectively. Overall, we observed that receptor-protein interaction substantially enhanced the number of hydrogen bonds, which was higher in the CTcS complex. The solvent-accessible surface area values changed owing to the binding of the ligand to the receptor (Figure 16). The reduced solvent-accessible surface area of the receptor upon binding to the ligand indicates the alteration of conformation in the protein structure and reduction in pocket size with increased hydrophobicity around it.

Overall, adding PA to Ris resulted in a higher binding affinity to serotonin compared to dopamine and adrenergic receptors. This finding, in the context of the known benefits of Ris, could enhance the therapeutic benefits of this compound in terms of improving side effects associated with lower Dopamine activity as well as higher affinity for serotonin receptors, which could aid in alleviating comorbid depression. Therefore, such findings could potentially bridge the literature gap in terms of designing a multiple receptor profile drug targeting the relevant receptors [11].

Previous animal studies showed that higher Ris affinity to dopamine receptors positively affects the molecular brain-to-plasma ratio [8]. Having a lower affinity for dopamine than that of serotonin receptors fits with study findings, which showed that higher dopamine affinity was associated with extrapyramidal side effects associated with first-generation antipsychotics [10]. The literature consensus regarding dopamine receptor affinity is to attain 70–80% binding, as any further binding could lead to unpleasant extrapyramidal side effects [10,37].

## 3. Materials and Methods

### 3.1. Synthesis of Six Ris Charge Transfer Complexes

The solid six risperidone solid charge transfer complexes with general formula [(Ris) (π–acceptor)] were produced as previously reported [26]. A total of 0.25 mmol of risperidone medication was dissolved in 20 mL methanol and reacted with 0.25 mmol of each acceptor; then, each mixture was stirred for 45 min at room temperature. The solid products were filtered out, washed with minimal quantities of chloroform, and dried under vacuum over anhydrous CaCl_2_.

### 3.2. Characterization

Structures of Ris and CT complexes, viz. [(Ris) (PA)], [(Ris) (DDQ)], [(Ris) (TCNQ)], [(Ris) (TCNE)], [(Ris) (BL)], and [(Ris) (CL)] were obtained in PDBQT format using OpenBabelIGUI software (version 2.4.1) [38]. The energy of the structures was then minimized by applying the MMFF94 force field and conjugate gradient optimization algorithm using PyRx-Python prescription 0.8 for 500 steps [39]. The 3D crystal structures of serotonin (PDB ID: 6BQH), dopamine (PDB ID: 6CM4), and adrenergic (PDB ID: 6KUW) receptors were retrieved from the RCSB Protein Data Bank [40]. Receptors were prepared by removing the native ligand and other heteroatoms, including water, using the BIOVIA Discovery Studio Visualizer (v19.1.0.18287). Kollman charges of the receptors were also determined, and polar hydrogen atoms were added using AutoDock Tool [41]. Partial charges were assigned using the Geistenger method. Autodock Vina [42] was used to perform the docking calculations. The resulting docked poses were analyzed using the DS Visualizer (https://www.3ds.com/products-services/biovia/) that was accessed on 1 December 2021. The overall docking experiment was run on a processor (Intel(R) Core(TM) i5-4200U CPU @ 1.60 GHz 2.10 GHz 2.30 GHz, 64-bit).

**Figure 17:**
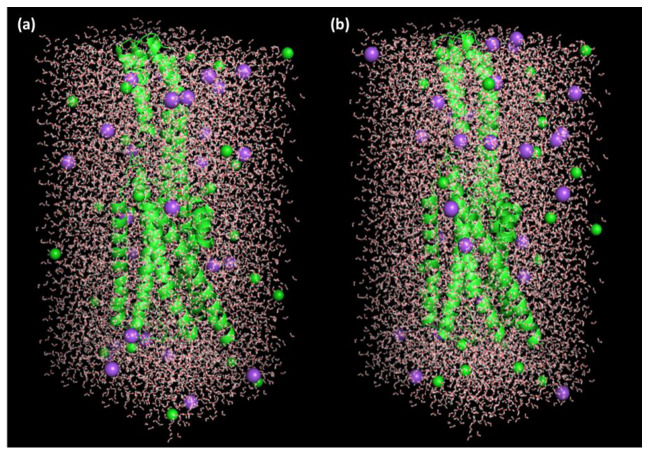
Receptor–ligand complexes, (**a**) RisS and (**b**) CTcS, in triclinic box solvated with water molecules and neutralized with 27 Na^+^ and 27 Cl^−^ ions (0.15 M salt).

For MD simulations and evaluation of their conformational space and inhibitory potential, the best receptor–ligand complex poses with the highest docking scores for Ris and the CT complexes obtained from molecular docking investigations were used. The Groningen Machine for Chemical Simulations (GROMACS) version 2019.2 package was used to perform MD simulation analysis with the GROMOS96 43a1 force field. The parameter files and topology of both ligands were generated using the latest CGenFF via CHARMM-GUI [43,44]. SPC water models that extended 10 Å from the protein were used to solve the protein–ligand structures in a triclinic box [45]. To mimic the physiological salt concentrations, 27 Na^+^ and 27 Cl^−^ ions (0.15 M salt) were added to neutralize the systems (Figure 17). In the NPT/NVT equilibration run, both systems were subjected to periodic boundary conditions at a constant temperature (300 K) and pressure (1.0 bar) for a 100 ns simulation duration using a Leap-frog MD integrator [46]. Energy minimization using the steepest descent approach with 5000 steps was used to eliminate bad contact inside the system [47]. Hydrogen bonding was examined using a gmx hbond tool. The gmx gyrate and gmx sasa tools were used to calculate the gyration radius and solvent-accessible surface area, respectively. Using gmx rms tools, the RMSD of the protein was computed. Trajectory analysis was performed using the GROMACS analysis tools [48]. Plots were prepared using Grace software version 5.1.21 and PyMol/VMD software version 2.0.2 [49,50,51] was used for visualization. Simulations were conducted using processor Intel(R) Xeon(R) CPU E5-2680 v4 @ 2.40 GHz, 64-bit.

## 4. Conclusions

Molecular docking revealed that the [(Ris) (PA)] CT complex interacted with all three receptors more efficiently than the reactant donor (Ris) did, and among all complex–receptor interactions, the CTcS combination had the highest binding energy. A MD simulation of the 100 ns run revealed that the RisS and CTcS complexes both possessed a stable conformation; however, CTcS formed a more stable complex with the serotonin receptor. Therefore, we present theoretical support for augmenting Ris with PA to enhance serotonergic receptor affinity, since lower binding affinity to dopamine receptors was observed compared with pure Ris. This enhancement of serotonergic binding according to the serotonin deficiency theory is believed to reduce the highly prevalent depressive episodes associated with depression. Therefore, these results could pave the way for the further optimization of risperidone for comorbid depression.

## Figures and Tables

**Table 1:** Docking scores of six synthesized CT complexes with three receptors.

**S. No.**	**Receptor**	**Binding Free Energy (kcal/mol)**						
		Ris	[(Ris) (PA)]	[(Ris) (BL)]	[(Ris) (CL)]	[(Ris) (DDQ)]	[(Ris) (TCNQ)]	[(Ris) (TCNE)]
1	Serotonin	−9.6	−11.4	−8.5	−9.0	−10.5	−10.0	−8.6
2	Dopamine	−8.4	−10.6	−9.8	−9.9	−10.0	−10.5	−8.8
3	Adrenergic	−9.1	−10.2	−10.2	−10.1	−9.8	−9.6	−8.5

**Table 2:** Docking scores of risperidone and its interactions with receptors.

S. No.	Receptor	Binding Free Energy (kcal/mol)	Interactions	
			H-Bond	Others
1	Serotonin	−9.6	Arg173	Leu325, Ala321, Val324 and Ala176 (π-Alkyl)
2	Dopamine	−8.4	His393	Val115, Phe389, Cys118, and Ile184 (π-Alkyl); Trp386 (π-Sigma)
3	Adrenergic	−9.1	Tyr427	Phe4155, Tyr405, and Leu204 (π-Alkyl)

**Table 3:** Docking scores of the CTcS complex and its interactions with receptors.

S. No.	Receptor	Binding Free Energy (kcal/mol)	Interactions	
			H-Bond	Others
1	Serotonin	−11.4	His182, Asn187, Asn384, Lys320 and Arg173	Leu325, Ala321, Ala108 and Ala176 (π-Alkyl); Asp172 (Halogen-fluorine)
2	Dopamine	−10.6	Thr142, Ala185, His393, and Tyr408	Val115 and Phe389 (π-Alkyl); Trp386 (π-Sigma); Cys118 (Halogen-fluorine)
3	Adrenergic	−10.2	Val414, Asp206, Asp131, and Ser218	Phe398, Phe423, and Cys135 (π-Alkyl); Val132 (π-Sigma); Ser214 (Halogen-fluorine)

## Data Availability

Data is contained within article.
